# Strong Correlation Between A‐Site Cation Order and Self‐Trapped Exciton Emission in 0D Hybrid Perovskites

**DOI:** 10.1002/smsc.202400443

**Published:** 2024-11-22

**Authors:** Feier Fang, Yongwang Shen, Yu Li, Kaimin Shih, Hanlin Hu, Haizhe Zhong, Yumeng Shi, Tom Tao Wu

**Affiliations:** ^1^ International Collaborative Laboratory of 2D Materials for Optoelectronics Science and Technology of Ministry of Education Institute of Microscale Optoelectronics Shenzhen University Shenzhen 518060 P. R. China; ^2^ Department of Applied Physics Hong Kong Polytechnic University Hung Hom Kowloon Hong Kong 999077 P. R. China; ^3^ Department of Computer Science and Engineering The Chinese University of Hong Kong Hong Kong SAR 999077 P. R. China; ^4^ The CUHK Shenzhen Research Institute Hi‐Tech Park, Nanshan Shenzhen 518057 P. R. China; ^5^ Department of Civil Engineering University of Hong Kong Pok Fu Lam Road Hong Kong SAR 999077 P. R. China; ^6^ Hoffmann Institute of Advanced Materials Shenzhen Polytechnic University 7098, Liuxian Boulevard Shenzhen 518055 P. R. China; ^7^ Key Laboratory of Luminescence and Optical Information Ministry of Education School of Physical Science and Engineering Beijing Jiaotong University Beijing 100044 P. R. China

**Keywords:** A‐site cations, order–disorder transformations, self‐trapped excitons, temperature sensings; 0D perovskites

## Abstract

Metal halide perovskites and their derived materials have garnered significant attention as promising materials for solar cell and light‐emitting applications. Among them, 0D perovskites, characterized by unique crystallographic/electronic structures with isolated metal halide octahedra, exhibit tremendous potential as light emitters with self‐trapped exciton (STE). However, the modulation of STE emission characteristics in 0D perovskites primarily focuses on regulating B‐ or X‐site elements. In this work, a lead‐free compound, Sb^3+^‐doped ((C_2_H_5_)_2_NH_2_)_3_InCl_6_ single crystal, which exhibits a high photoluminescence quantum yield, is synthesized, and with increasing temperature, the A‐site organic cations undergo a transition from an ordered configuration to a disordered one, accompanied by a redshift in the STE emission. Furthermore, Hirshfeld surface calculations reveal that high temperatures enhance the thermal vibrations of SbCl_6_^3−^ clusters and the octahedra distortion, which are responsible for the redshift. Since this thermally triggered transition of A‐site order is reversible, it can be exploited for temperature‐sensing applications. Overall, in this work, valuable insights are provided into the role of A‐site cations in modulating STE emission and the design of efficient light emitters.

## Graphical abstract

In 0D perovskite Sb^3+^‐doped ((C_2_H_5_)_2_NH_2_)_3_InCl_6_, the A‐site organic cations undergo a transition from an ordered configuration to a disordered one as the temperature increases. This transition enhances the thermal vibrations of SbCl_6_^3−^ clusters and the octahedra distortion, which results in color change, i.e., redshift of wavelength, in the self‐trapped exciton emission.

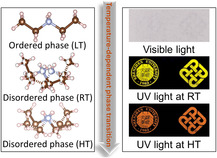

## 1 Introduction

Metal halide perovskites have garnered significant interest due to their excellent optoelectronic properties.^[^ ^1^, ^2^, ^3^, ^4^ ^]^ Organic–inorganic hybrid metal halide perovskites demonstrate remarkable structural tunability relative to their purely inorganic counterparts, attributable to the wide variety of organic and inorganic constituents that can be integrated into their framework. Unlike 3D metal halides, low‐dimensional compositions generally possess localized transient elastic distortions of the lattice due to strong electron–phonon coupling, often leading to the formation of novel excitation states such as self‐trapped excitons (STEs).^[^ ^5^, ^6^, ^7^, ^8^, ^9^, ^10^, ^11^, ^12^ ^]^ Further research has revealed that the vibrational degrees of freedom in halide perovskites are enhanced as the dimensional structure decreases, thereby favoring the formation of STEs.^[^ ^13^, ^14^, ^15^ ^]^ Among the great family of organic–inorganic metal halide perovskites, 0D perovskites have attracted significant attention due to their retention of photophysical characteristics of individual metal halide octahedra or metal halide clusters.^[^ ^16^ ^]^ Additionally, they exhibit broadband emissions with significant Stokes shift and high photoluminescence (PL) quantum yield (PLQY) originating from STEs.^[^ ^17^, ^18^, ^19^, ^20^ ^]^

Currently, research on the modulation of emission properties in 0D perovskites primarily focuses on altering the composition of B‐ or X‐site elements, as the atomic orbitals of B/X site ions directly participate in the formation of the conduction and valence band edges, thereby affecting the luminescence performance.^[^ ^21^ ^]^ In contrast, regulating the luminescent properties of halide perovskites through the A‐site composition has received comparatively less research attention. Generally, the A cations do not directly contribute to the formation of the optical bandgap, but they still have a significant impact on the optical properties by dictating the tilting of BX_6_ octahedra and the structural distortions.^[^ ^21^, ^22^, ^23^ ^]^ Particularly, the STE emission is greatly affected by the lattice softness.^[^ ^24^, ^25^, ^26^, ^27^ ^]^ In rigid structures, such as Sb^3+^‐doped oxides and double perovskites, the distortion of SbCl_6_^3−^ polyhedron is limited, leading to a relatively short‐wavelength emission.^[^ ^28^ ^]^ However, in soft structures such as hybrid metal halides, the SbCl_6_^3−^ polyhedron undergoes significant distortion in a more relaxed environment, resulting in a significant Stokes shift.^[^ ^29^, ^30^, ^31^ ^]^ In our previous work,^[^ ^32^ ^]^ the contribution of polar A‐site cations to *E*_Stokes_ was experimentally demonstrated by incorporating polar A‐site cation OH_3_^+^ into the perovskite structure. Recently, a series of 0D indium (In)‐based organic–inorganic metal halide perovskites were synthesized with different A‐site cations.^[^ ^32^, ^33^ ^]^ By incorporating antimony ions, the researchers discovered a significant correlation between the degree of distortion of polyhedra associated with A‐site cations and STE emission.^[^ ^32^, ^33^ ^]^ However, to the best of our knowledge, the influence of varying A‐site cations within the same compound on STE emission has not been reported.

In this work, we investigated Sb^3+^‐doped ((C_2_H_5_)_2_NH_2_)_3_InCl_6_, which has a unique organic cation ((C_2_H_5_)_2_NH_2_)^+^, as a prototype, to offer insights into the correlation between the octahedral distortion and light emission in organic–inorganic hybrid metal halide perovskites. At low temperatures (LT), the ((C_2_H_5_)_2_NH_2_)^+^ cations exhibit a highly ordered state with well‐defined positions of the hydrogen (H) atoms and strong hydrogen bonding. As the temperature increases, the dynamic disorder of the organic cation and the consequent changes in the hydrogen bonding are accompanied by significant changes in the tilting of the inorganic octahedra, resulting in a redshift of the STE emission. Considering the high reversibility of this thermally induced transformation, we utilized Sb^3+^‐doped ((C_2_H_5_)_2_NH_2_)_3_InCl_6_ as an ink in anticounterfeiting strategies, achieving multiple encryption effects. This work provides valuable insights into the effect of A‐site cation order on the STE emission of halide perovskites and promotes the application of this emerging class of light‐emitting materials.

## 2 Results and Discussion

**Figure 1:**
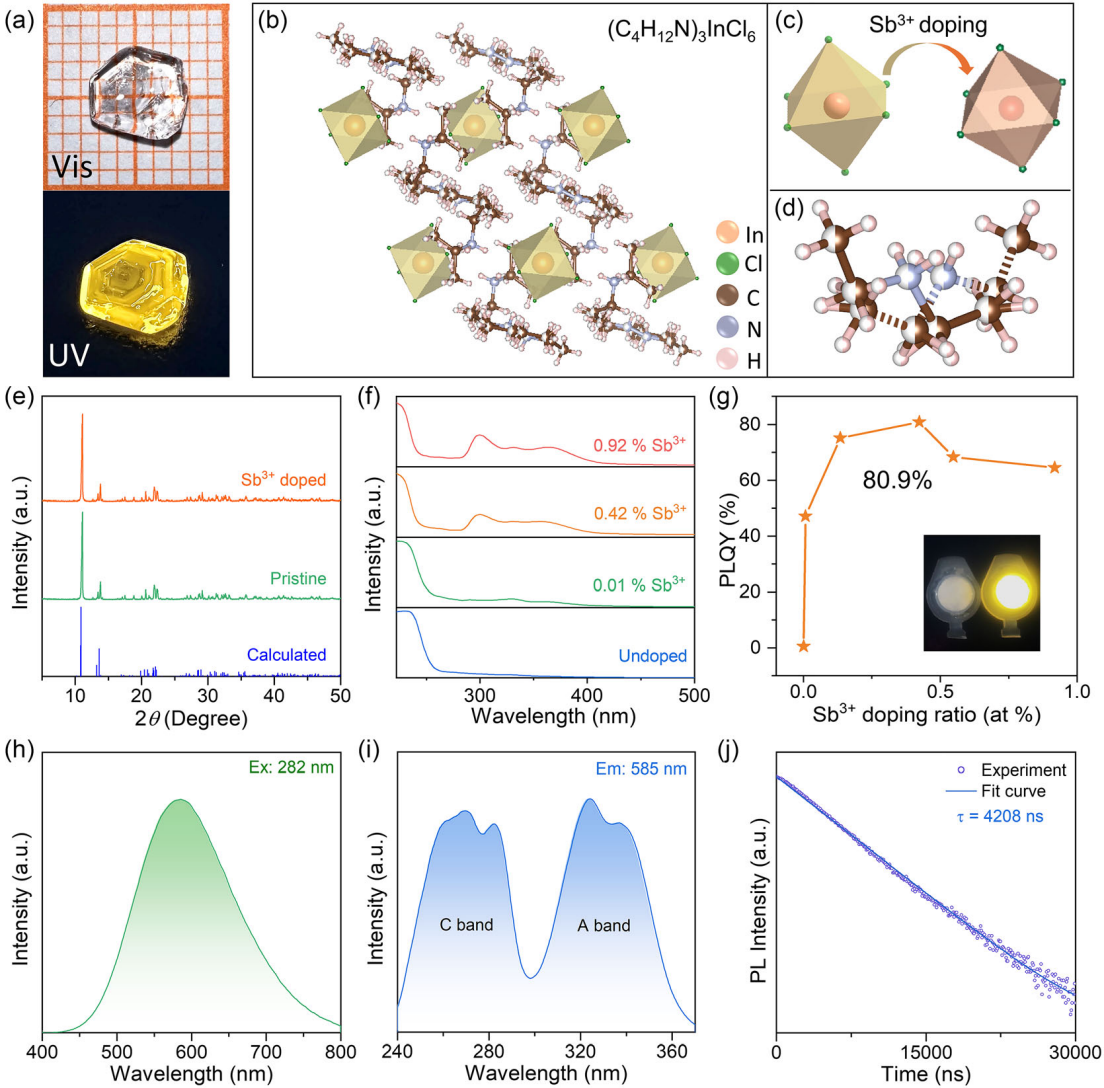
a) Images of the Sb^3+^‐doped ((C_2_H_5_)_2_NH_2_)_3_InCl_6_ single crystal upon visible light (top) and 302 nm ultraviolet light (bottom) illumination. b) Crystal structure of the hybrid 0D ((C_2_H_5_)_2_NH_2_)_3_InCl_6_. c) Schematic diagram illustrating the Sb^3+^ substitution. d) Structure and configuration of the ((C_2_H_5_)_2_NH_2_)^+^ cations at room temperature. e) Powder X‐Ray diffraction (PXRD) patterns of hybrid 0D ((C_2_H_5_)_2_NH_2_)_3_InCl_6_ with and without Sb^3+^ doping. f) Ultraviolet−visible absorption spectra of hybrid 0D ((C_2_H_5_)_2_NH_2_)_3_InCl_6_ with increased Sb^3+^ dopant levels. g) PLQY of ((C_2_H_5_)_2_NH_2_)_3_InCl_6_:xSb^3+^ with different Sb^3+^ levels. The inset shows photograph of undoped ((C_2_H_5_)_2_NH_2_)_3_InCl_6_ (left) and ((C_2_H_5_)_2_NH_2_)_3_InCl_6_:0.42 Sb^3+^ (right) single crystals under UV light. h) Photoluminescence (PL) spectra of Sb^3+^‐doped ((C_2_H_5_)_2_NH_2_)_3_InCl_6_ excited at 282 nm. i) Photoluminescence excitation (PLE) spectra of Sb^3+^‐doped ((C_2_H_5_)_2_NH_2_)_3_InCl_6_ emission at 585 nm. j) PL decay and fitting curve of Sb^3+^‐doped ((C_2_H_5_)_2_NH_2_)_3_InCl_6_ at room temperature (RT).

Sb^3+^‐doped ((C_2_H_5_)_2_NH_2_)_3_InCl_6_ single crystals (SCs) were obtained through a slow evaporation crystallization process from an ethanol solution (see details in Supporting Information). As shown in **Figure** **1**a, the plate‐shaped crystal appears colorless and transparent under daylight, but when exposed to UV light with a wavelength of 302 nm, it yields bright yellow light. To determine the crystal structure, SC X‐Ray diffraction (SCXRD) analysis was performed, which yielded SC fitting with satisfactory accuracy (*R*_1_ = 0.0486, w*R*_2_ = 0.1185, *S* = 1.045, Table S1, Supporting Information). The crystal exhibits monoclinic space group C2/c, with cell parameters *a* = 18.3 Å, *b* = 10.2 Å, and *c* = 14.3 Å. At the molecular level, the In–Cl polyhedrons are isolated with ((C_2_H_5_)_2_NH_2_)^+^ cations in between, thus forming a 0D structure (Figure 1b). As shown in Figure 1c, Sb^3+^ ions occupy the In^3+^ sites to form SbCl_6_^3−^ octahedrons, similar to the pure inorganic Cs_3_InCl_6_:Sb^3+^ system.^[^ ^34^ ^]^ Figure 1d and S1, Supporting Information, show the configurations of the ((C_2_H_5_)_2_NH_2_)^+^ cation, and the disorder can be attributed to the large freedom of motion of this organic molecule.^[^ ^35^, ^36^ ^]^ As shown in Figure 1e, the structure of the Sb^3+^‐doped sample was characterized using powder X‐Ray diffraction (PXRD), which revealed that samples with various Sb^3+^ doping concentrations (0.01–0.92%) retained the ((C_2_H_5_)_2_NH_2_)_3_InCl_6_ crystal structure. The incorporation of Sb^3+^ had a minimal impact on the original structure, which can be attributed to the similar ionic radii of Sb^3+^ (76 pm) and In^3+^ (80 pm).^[^ ^37^, ^38^ ^]^ Figure S2, Supporting Information, shows the results of energy‐dispersive spectral mapping, revealing the homogeneous distribution of Sb^3+^ dopants within the ((C_2_H_5_)_2_NH_2_)_3_InCl_6_ crystal.

Absorption spectroscopy was utilized to investigate the optical properties of the samples. It is recognized that Sb^3+^ dopants serve as color centers in In‐based perovskite hosts.^[^ ^24^ ^]^ As shown in Figure 1f, the characteristic absorption peaks associated with Sb^3+^ have a positive correlation with the Sb^3+^ doping ratio, indicating that the presence of Sb^3+^ ions enhances the light absorption of the samples. As shown in Figure S3 and Table S2, Supporting Information, the inductively coupled plasma optical emission spectroscopy was employed to determine the doping ratio of Sb^3+^. The results demonstrated a linear relationship between the actual amount of Sb^3+^ and the feed ratio, suggesting that the doping ratio of Sb^3+^ can be effectively controlled during the crystal growth process.

As shown in Figure 1g, the concentration of Sb^3+^ dopant has a significant impact on the PLQY. By increasing the concentration of Sb^3+^ dopants, the PLQY significantly improves from 0.5% to 80.9% (Figure S4, Supporting Information). However, a further increase in the amount of Sb^3+^ doping results in lower PLQY due to the concentration‐related quenching.^[^ ^39^, ^40^ ^]^ Samples with different concentrations of Sb^3+^ doping exhibit similar PL peaks, as shown in Figure S5, Supporting Information. Therefore, the sample with a 0.42% Sb^3+^ doping concentration, which exhibits the maximum PLQY value, was selected for further analysis. The Sb^3+^‐doped ((C_2_H_5_)_2_NH_2_)_3_InCl_6_ crystal exhibits bright broadband PL emission at 585 nm with a full width at half maximum (FWHM) of ≈165 nm (Figure 1h). Due to the spatial confinement of the 0D structure, Sb^3+^ ions undergo a strong Jahn–Teller distortion in the excited state,^[^ ^13^, ^41^ ^]^ resulting in a large Stokes shift of ≈303 nm. Figure 1i depicts the PLE fine structure observed in the sample, which originates from the ns^2^ electronic configuration.^[^ ^42^ ^]^ According to the previous report,^[^ ^42^ ^]^ the double excitation bands observed at 324 and 338 nm can be assigned to the ^1^S_0_ → ^3^P_1_ transition of Sb^3+^ ions, commonly denoted as the A band. Additionally, the three excitation bands at 258, 270, and 282 nm can be attributed to the ^1^S_0_ → ^1^P_1_ transition, named as the C band.^[^ ^42^, ^43^, ^44^ ^]^ To reveal the underlying mechanism of the broadband emission, time‐resolved PL spectra were measured. As shown in Figure 1j, the PL decay curve of Sb^3+^‐doped ((C_2_H_5_)_2_NH_2_)_3_InCl_6_ can be well fitted by a single‐exponential function, with an estimated lifetime of 4.2 μs. The overall PL characteristics, including broad emission, large Stokes shift, and microsecond‐scale lifetime, indicate that the broadband yellow emission originates from the radiative recombination of STEs associated with Sb^3+^ ions.^[^ ^13^, ^30^, ^31^ ^]^

**Figure 2:**
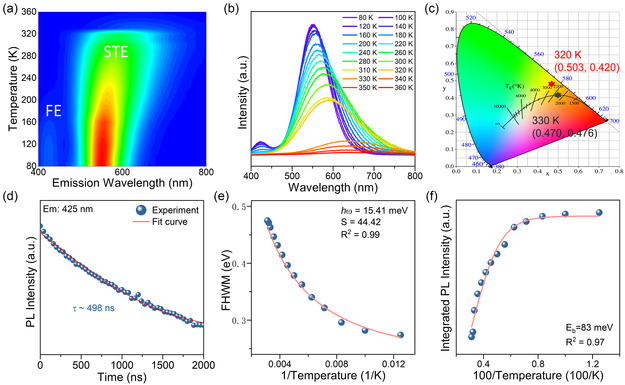
a) Mapping of PL spectra of Sb^3+^‐doped ((C_2_H_5_)_2_NH_2_)_3_InCl_6_ at various temperatures from 80 to 360 K. b) Temperature‐dependent PL spectra collected from 80 to 360 K. c) The Commission Internationale de l’Eclairage (CIE) chromaticity coordinates for ((C_2_H_5_)_2_NH_2_)_3_InCl_6_:0.42%Sb^3+^ at 320 K (red pentagram) and 330 K (black pentagram). d) Time‐resolved PL decay curve collected on the sample at 80 K. e) FWHM of the PL spectra measured from 80 to 320 K. f) Integrated PL intensity as a function of 100/T.

To gain further insights into the luminescence mechanism, temperature‐dependent PL measurements were conducted on Sb^3+^‐doped ((C_2_H_5_)_2_NH_2_)_3_InCl_6_ in the temperature range of 80–360 K. As shown in **Figure** **2**a, the PL emission peak exhibits a consistent redshift as the temperature increases. Notably, Figure 2b reveals an abrupt shift in the peak position when the temperature increases from 320 to 330 K. As shown in Figure S6, Supporting Information, there is a pronounced redshift in the emission maxima from 585 to 640 nm, resulting in a corresponding transition from yellow to orange emission. This observation is further supported by the plot of CIE chromaticity coordinates, as shown in Figure 2c. The distinct thermally triggered changes in PL behavior hint at a thermodynamic structural phase transformation, which is evident in Figure S7, Supporting Information, of the PLE spectra as well. A detailed discussion will be provided in the following section.

Meanwhile, upon cooling down to 80 K, a distinct PL component at ≈425 nm was observed. To investigate the nature of the LT light emission, transient PL measurement was carried out for Sb^3+^‐doped ((C_2_H_5_)_2_NH_2_)_3_InCl_6_. As shown in Figure 2d, the sample exhibited a fast decay lifetime of 498 ns, which is much shorter than the STE emission and can be attributed to free excitons. As shown in Figure 2e, the temperature‐dependent PL intensity could be fitted using the following equation

(1) $$\begin{array}{c} \mathrm{FWHM}=2.36\sqrt{S}\hbar \omega_{\text{phonon}}\sqrt{\text{coth}\frac{\hbar \omega_{\text{phonon}}}{2k_{B}T}} \end{array}$$

where *ħ* is the reduced Planck constant, *S* is the Huang–Rhys factor, *ω*_phonon_ is the phonon frequency, and *T* is the temperature.^[^ ^45^, ^46^, ^47^ ^]^ The general increase of FWHM with temperature can be attributed to the enhanced electron–phonon coupling. As shown in Figure S8, Supporting Information, owing to the PL transition, only the FWHM data obtained from 80 to 320 K could be fitted well. The Huang–Rhys factor *S* is associated with the intensity of the electron–phonon coupling. Generally, a larger *S* factor is considered beneficial for the formation of STEs. Using the aforementioned equation, the *S* factor was calculated to be 44.42, indicating a strong electron–phonon coupling in the material system, which is conducive to the formation of STEs.^[^ ^13^, ^14^, ^15^ ^]^

The exciton binding energy is closely associated with the radiative recombination process, which can be fitted by using the following equation^[^ ^46^, ^48^ ^]^

(2) $$\begin{array}{c} I\left(T\right)=\frac{I\left(0\right)}{1+A\exp\left(-\frac{E_{b}}{k_{B}T}\right)} \end{array}$$

where *I*(*T*) is the PL integrated intensity, *I*(0) is the PL intensity at 0 K, *E*_b_ is the exciton binding energy, and *k*_B_ is the Boltzmann constant. As shown in Figure 2f, the fitting to the data from 80 to 320 K yields an *E*_b_ of 83 meV for Sb^3+^‐doped ((C_2_H_5_)_2_NH_2_)_3_InCl_6_. The value of *E*_b_ is higher than the thermal energy at RT (≈26 meV), which is beneficial for charge trapping and the formation of STEs.^[^ ^25^, ^49^ ^]^

**Figure 3:**
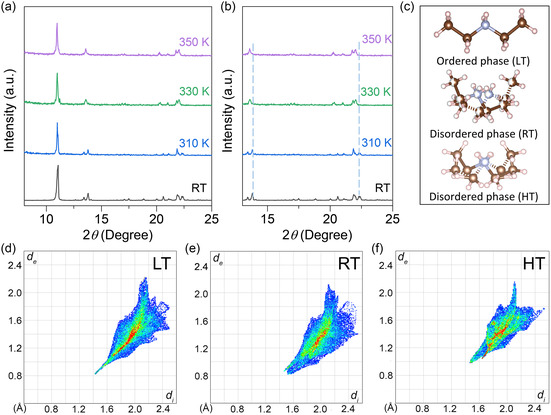
a) Temperature‐dependent XRD patterns of ((C_2_H_5_)_2_NH_2_)_3_InCl_6_. b) XRD patterns between 13° and 25°. c) Crystal structure of the ((C_2_H_5_)_2_NH_2_)^+^ units at different temperatures. The 2D Hirshfeld fingerprint plots for SbCl_6_^3−^ at d) LT (134 K), e) RT (296 K), and f) HT (330 K). The more intense color means a stronger interaction between SbCl_6_^3−^ and the organic cations.

To confirm the existence of a phase transition in ((C_2_H_5_)_2_NH_2_)_3_InCl_6_, we conducted PXRD at various temperatures (300, 310, 330, and 350 K), as shown in **Figure** **3**a,b. The results revealed distinct variations between the room temperature and high temperature (RT and HT) patterns, thus providing compelling evidence for a phase transition in ((C_2_H_5_)_2_NH_2_)_3_InCl_6_. As shown in Figure S9, Supporting Information, the peak at around 11.08° slightly shifts to a lower diffraction angle as the temperature increases, suggesting lattice expansion.^[^ ^50^ ^]^ Notably, two diffraction peaks at 310 K (around 13.79° and 22.35°) are replaced by significantly stronger diffraction peaks at 330 K (around 13.57° and 22.05°), implying a structural phase transition in the crystal. Moreover, it is worth noting that the profile patterns recorded in the RT phase, before the phase transition and upon cooling down back to the RT phase from the HT phase, remain unchanged. This observation strongly supports the occurrence of a reversible phase transition in ((C_2_H_5_)_2_NH_2_)_3_InCl_6_, which aligns with the differential scanning calorimetry (DSC) results (Figure S10, Supporting Information). The crystal structure of the 0D ((C_2_H_5_)_2_NH_2_)_3_InCl_6_ SC at LT, RT, and HT was determined using SCXRD, as shown in Figure S11 and Table S1, S3, and S4, Supporting Information. At RT, the sample crystallized in the monoclinic crystal system with a *C*2/*c* space group and cell parameters of *a* = 18.32 Å, *b* = 10.25 Å, and *c* = 14.29 Å. At HT, the sample transitions into the triclinic crystal system with an *R‐3c* space group and cell parameters of *a* = 10.52 Å, *b* = 10.52 Å, and *c* = 38.79 Å. Compared to the unit volume of the crystal at RT, the unit volume at HT increased by ≈1.5 times, and the cations became disordered at these two states. The changes of lattice symmetry and volume reflect the average states of the atomic‐level disordering.^[^ ^51^ ^]^ Upon reducing the temperature to LT, the disordered C and N atoms froze, and the LT structure became completely ordered. Consequently, all atoms possess their exclusive positions, as illustrated in Figure 3c. According to the previous reports, the order–disorder transformation of the organic cations significantly influences the alignment and distortion of the inorganic octahedra, which in turn affects the STE emission.^[^ ^21^, ^52^, ^53^, ^54^ ^]^

To analyze the interaction between the SbCl_6_^3−^ and the organic cations at different temperatures, Cl–H interactions were analyzed using Hirshfeld surface calculation.^[^ ^55^ ^]^ In the 2D fingerprint plots, the red‐colored area indicates a strong Cl–H interaction (i.e., the interaction between the anion and cation), while the blue‐colored area refers to a weak one.^[^ ^55^, ^56^, ^57^ ^]^ As shown in Figure 3d–f, SbCl_6_^3−^ has a stronger interaction with organic cations at LT than that at RT and HT. Moreover, the shorter distances of the spikes at the bottom left of the fingerprint plots with the coordinate origins indicate stronger hydrogen bonding.^[^ ^58^ ^]^ As a result, the motion freedom of SbCl_6_^3−^ at HT is much higher than that at LT and RT. The soft structure of Sb^3+^‐doped ((C_2_H_5_)_2_NH_2_)_3_InCl_6_ at HT increases the thermal vibration and SbCl_6_^3−^ octahedral distortion. Therefore, these data provide insights into the transformation of ((C_2_H_5_)_2_NH_2_)^+^ cations from order to disorder, which significantly alters the configurations of the inorganic octahedra, leading to the observed redshift of the STE emission.

**Figure 4:**
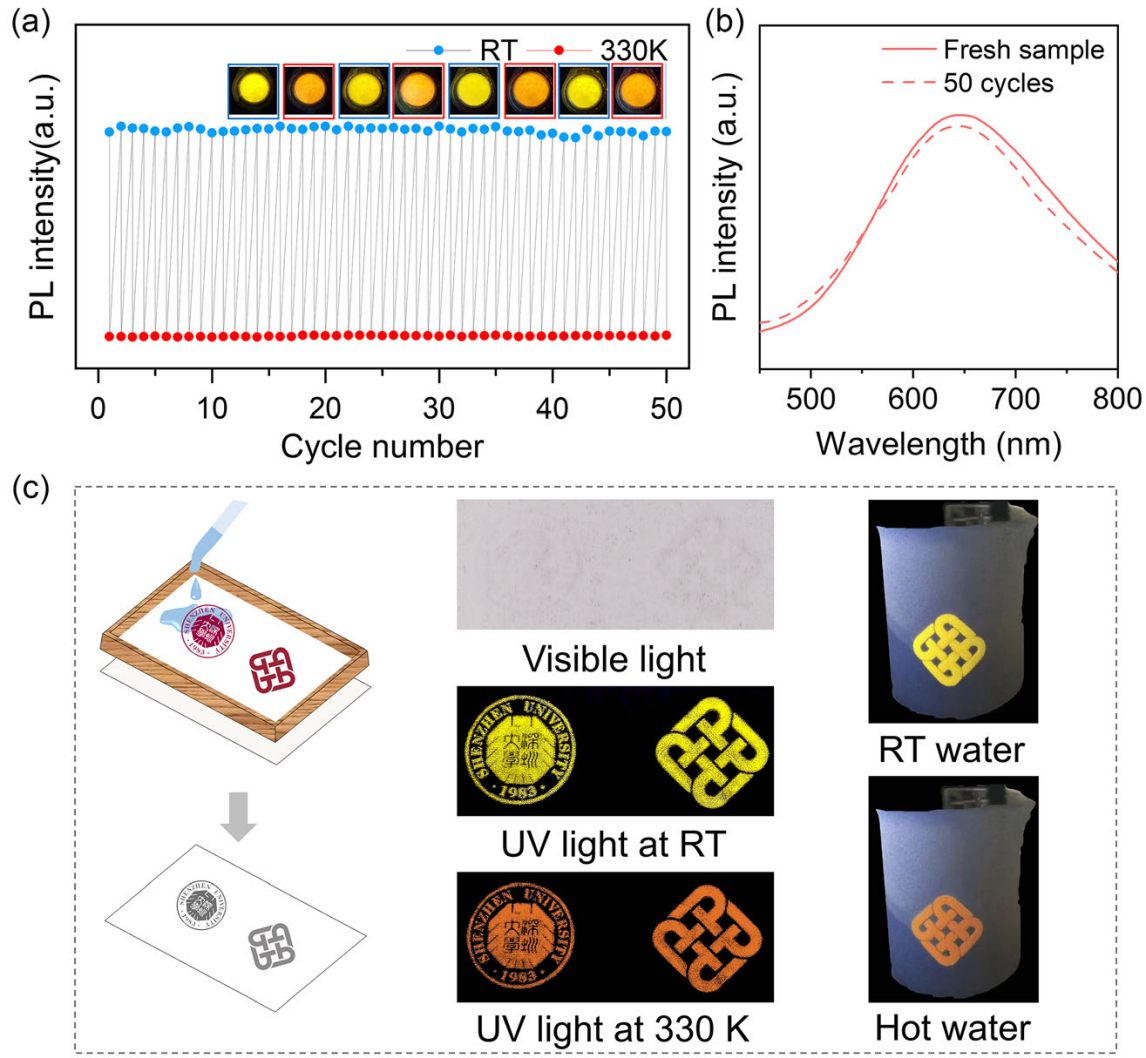
a) PL intensity retention over 50 cycles, with a vacuum chamber used to eliminate the effects of moisture. The inset shows photographs of the Sb^3+^‐doped ((C_2_H_5_)_2_NH_2_)_3_InCl_6_ sample at RT (blue box) and 330 K (red box) when exposed to UV light at 302 nm. b) Comparison of the PL emission of the initial powder and the one after 50 heating and cooling cycles. c) Schematic diagram of screen printing (left) and printed patterns revealed using the Sb^3+^‐doped ((C_2_H_5_)_2_NH_2_)_3_InCl_6_ precursor solutions on nonwoven fabrics exposed to visible light and images of the printed patterns at RT and 330 K under 302 nm UV light. (Middle) Images of the printed patterns at RT water and hot water under 302 nm UV light (right).

The cycling performance of temperature‐dependent PL spectra is shown in **Figure** **4**a. As depicted in Video S1, Supporting Information, the emission color of Sb^3+^‐doped ((C_2_H_5_)_2_NH_2_)_3_InCl_6_ reversibly changed between yellow and orange when the temperature altered between RT and 330 K, and the complete transition takes less than 2 min. Remarkably, after 50 cycles, the emission intensity fully returns to the initial level, with no significant changes in the PL spectra, as depicted in Figure 4a,b. The significant temperature‐induced modulation of the emission wavelength of Sb^3+^‐doped ((C_2_H_5_)_2_NH_2_)_3_InCl_6_ and its unique reversible conversion characteristics render it a promising material for temperature‐dependent sensing and anticounterfeiting.

Herein, anticounterfeiting application was demonstrated based on the temperature‐induced conversion of Sb^3+^‐doped ((C_2_H_5_)_2_NH_2_)_3_InCl_6_. Figure 4c depicts a schematic illustrating the utilization of Sb^3+^‐doped ((C_2_H_5_)_2_NH_2_)_3_InCl_6_ precursor solutions as security inks in screen printing. As a proof of concept to demonstrate the double encryption, various patterns were drawn on a nonwoven fabric, as shown in Figure 4c. These patterns were nearly invisible under visible‐light irradiation at RT, while under UV light (302 nm) at RT, the encrypted pattern becomes visible and exhibits yellow emission. When the temperature is raised to 330 K, the encrypted pattern exhibits orange emission under 302 nm UV light. The fast and repeatable temperature‐dependent color conversion of Sb^3+^‐doped ((C_2_H_5_)_2_NH_2_)_3_InCl_6_ could dramatically improve the authenticity of labels and facilitate the establishment of a multilevel encryption.

Notably, the World Health Organization recommends maintaining the temperature of drinking water below 333.15 K (≈60 °C) to prevent scorching and burns. Based on the DSC results, the phase transition temperature of Sb^3+^‐doped ((C_2_H_5_)_2_NH_2_)_3_InCl_6_ was estimated to be ≈326.6 K, which is close to 333.15 K. Therefore, such thermochromic fluorescent materials integrated into water cups can serve as visual indicators of water with temperatures suitable for consumption. As illustrated in Figure 4c, when the cup contains hot water, the material shows orange fluorescence, signaling the risk of scalding and a need for caution. However, as the water temperature gradually decreases to RT, the fluorescence color changes to yellow, indicating the suitability of the water sample for consumption without the risk of scalding.

## 3 Conclusion

In summary, a 0D organic–inorganic metal halide ((C_2_H_5_)_2_NH_2_)_3_InCl_6_ was successfully synthesized. Upon doping with Sb^3+^ ions, the samples exhibit broadband yellow emission centered at 585 nm with a PLQY of 80.9% at RT. The arrangement of ((C_2_H_5_)_2_NH_2_)^+^ cations converts from an ordered state to a disordered one with increasing temperature. Meanwhile, the transition is accompanied by significant changes in the tilting of the inorganic octahedra, resulting in a redshift in the STE emission. According to the Hirshfeld surface calculations, the interactions between SbCl_6_^3−^ ions and organic cations are weaker at HT and RT compared with those at LT. These weaker interactions amplify the thermal vibrations of SbCl_6_^3−^ ions and increase the likelihood of SbCl_6_^3−^ octahedral distortion. The reversibility of this transition facilitates the application of Sb^3+^‐doped ((C_2_H_5_)_2_NH_2_)_3_InCl_6_ materials as inks for anticounterfeiting applications, enabling multiple encryption effects. Overall, our findings offer valuable insights into the effects of A‐site cations on the STE emission characteristics of halide perovskites and facilitate the design of high‐performance light‐emitting materials.

## Conflict of Interest

The authors declare no conflict of interest.

## Author Contributions

**Feier** **Fang**: Conceptualization: (lead); Data curation: (equal); Formal analysis: (lead); Writing—original draft: (lead). **Yongwang** **Shen**: Data curation: (equal); Investigation: (lead). **Yu** **Li**: Resources: (supporting). **Kaimin** **Shih**: Resources: (supporting). **Hanlin** **Hu**: Resources: (supporting). **Haizhe** **Zhong**: Project administration: (equal); Supervision: (lead). **Yumeng** **Shi**: Funding acquisition: (lead); Supervision: (lead); Writing—review & editing: (lead). **Tom** **Tao** **Wu**: Funding acquisition: (lead); Project administration: (lead); Supervision: (lead); Writing—review & editing: (lead). **Feier Fang** and **Yongwang Shen** contributed equally to this work.

## Supporting information

Supplementary Material

## Data Availability

The data that support the findings of this study are available in the supplementary material of this article.
