# Supplementary material for: Strong Correlation Between A‐Site Cation Order and Self‐Trapped Exciton Emission in 0D Hybrid Perovskites
Source: Small Sci. 2024 Nov 22;5(2):2400443. doi: 10.1002/smsc.202400443 (PMC11934909; doi:10.1002/smsc.202400443)
Supplement: Supplementary file 1 — Supplementary Material [file SMSC-5-2400443-s001.zip › SI_submit.docx]

**Strong Correlation between A-Site Cation Order and Self-Trapped Exciton Emission in Zero-Dimensional Perovskites**

Feier Fang,^[a, b]ǂ^ Yongwang Shen,^[a]ǂ^ Yu Li,^[c, d]^ Kaimin Shih,^[e]^ Hanlin Hu,^[f]^ Haizhe Zhong,^[a]*^ Yumeng Shi,^[g]*^ Tom Wu^[b]*^

[a] International Collaborative Laboratory of 2D Materials for Optoelectronics Science and Technology of Ministry of Education, Institute of Microscale Optoelectronics, Shenzhen University, Shenzhen 518060, P. R. China.

[b] Department of Applied Physics, Hong Kong Polytechnic University, Hung Hom, Kowloon, Hong Kong, China.

[c] Department of Computer Science and Engineering, The Chinese University of Hong Kong, Hong Kong SAR, China

[d] The CUHK Shenzhen Research Institute, Hi-Tech Park, Nanshan, Shenzhen, 518057, China

[e] Department of Civil Engineering, University of Hong Kong, Pok Fu Lam Road, Hong Kong Special Administrative Region of China

[f] Hoffmann Institute of Advanced Materials, Shenzhen Polytechnic University, 7098 Liuxian Boulevard, Shenzhen 518055, People’s Republic of China

[g] Key Laboratory of Luminescence and Optical Information, Ministry of Education, School of Physical Science and Engineering, Beijing Jiaotong University, Beijing 100044, China

**Experimental Section**

**Materials**

Diethylaminehydrochloride (AR,99%metals basis), indium chloride (InCl_3_; anhydrous, 99.99% metals basis) and antimony trichloride (SbCl_3_, 99.9% metal basis) was purchased from Aladdin, and Ethanol (EtOH; 99% analytical reagent) was purchased from Xilong Chemistry (Guangdong, China). All the precursors and solvents were used without further purification.

**Synthesis of** **((C_2_H_5_)_2_NH_2_)_3_InCl_6_ and Sb^3+^-doped ((C_2_H_5_)_2_NH_2_)_3_InCl_6_ single crystals**

To prepare the single crystals, 15 mmol diethylaminehydrochloridea and 5 mmol InCl_3_ were dissolved in 2 mL of EtOH solution. Next, high-quality single crystals were obtained by slowly evaporating the solvent at room temperature for a few days. Similarly, to obtain the Sb^3+^-doped crystals, varying amounts of SbCl_3_ were added to the mixed powders of diethylamine hydrochloride (15 mmol), InCl_3_(5 mmol) to vials of EtOH (2 mL).

**Characterization**

The powder X-ray diffraction (PXRD) measurements of Sb^3+^-doped ((C_2_H_5_)_2_NH_2_)_3_InCl_6_ samples were performed using a D2 diffractometer (Bruker, Billerica, MA, USA) with Cu Kα radiation (λ = 1.5418 Å). The optical UV−vis absorption spectra were collected by a UV–vis–near-infrared (NIR) spectrometer (UV-2600i, Shimadzu, Kyoto, Japan). The room-temperature PL, PL excitation (PLE) spectra were measured using a fluorescence spectrophotometer (FS5, Edinburgh Instruments, Livingston, UK). Time-resolved PL (TRPL) spectroscopy measurements were also performed using the FS5 fluorescence spectrometer with a 283-nm laser as the excitation source. The temperature-dependent spectra were also collected by this fluorescence spectrometer. Inductively coupled plasma mass spectrometry (ICP-MS) was performed using an atomic emission spectrometer (Agilent 7700).

**Table S1. Single crystal X-ray diffraction data of ((C_2_H_5_)_2_NH_2_)_3_InCl_6_ single crystals at room temperature.**

| **Compound** | **((C_2_H_5_)_2_NH_2_)_3_InCl_6_** |
| --- | --- |
| Temperature(K) | 296 |
| Crystal system | monoclinic |
| Space group | C 2/c |
| a(Å) | 18.3186(11) |
| b(Å) | 10.2477(5) |
| c(Å) | 14.2866(7) |
| α° | 90 |
| β° | 116.538（1） |
| γ° | 90 |
| Volume(Å^3^) | 2399.4(2) |
| Z | 4 |
| ρ_calc_ (g/cm^3^) | 1.522 |
| μ (mm^-1^) | 1.653 |
| F (000) | 1120.0 |
| Theta(max) | 26.372 |
| R(reflections) | 0.0486(1889) |
| wR2(reflections) | 0.1185（2451） |
| no. of parameters | 148 |
| S | 1.045 |

**Table S2.** Feeding concentration and real Sb^3+^ atomic ratio in In-Based ((C_2_H_5_)_2_NH_2_)_3_InCl**_6_** measured by ICP-OES.

| **Sb^3+^-feeding concentration (%)** | **Sb^3+^ atomic ratio (%)** |
| --- | --- |
| 0 | 0.0003 |
| 0.2 | 0.0072 |
| 1 | 0.1351 |
| 2 | 0.4236 |
| 4 | 0.5482 |
| 6 | 0.9186 |

| **Compound** | **((C_2_H_5_)_2_NH_2_)_3_InCl_6_** |
| --- | --- |
| Temperature(K) | 134 K |
| Crystal system | monoclinic |
| Space group | P 21/c |
| a(Å) | 17.382(4) |
| b(Å) | 10.170(2) |
| c(Å) | 14.199(3) |
| α° | 90 |
| β° | 110.405(7) |
| γ° | 90 |
| Volume(Å^3^) | 2352.5(9) |
| Z | 4 |
| ρ_calc_ (g/cm^3^) | 1.553 |
| μ (mm^-1^) | 14.293 |
| F (000) | 1120.0 |
| Theta(max) | 79.729 |
| R(reflections) | 0.0221(4480) |
| wR2(reflections) | 0.0602(4869) |
| no. of parameters | 209 |
| S | 1.087 |

**Table S3. Single crystal X-ray diffraction data of ((C_2_H_5_)_2_NH_2_)_3_InCl_6_ single crystals at 134 K.**

| **Compound** | **((C_2_H_5_)_2_NH_2_)_3_InCl_6_** |
| --- | --- |
| Temperature(K) | 330 |
| Crystal system | hexagonal |
| Space group | R-3c |
| a(Å) | 10.5184(6) |
| b(Å) | 10.5184(6) |
| c(Å) | 38.790(4) |
| α° | 90 |
| β° | 90 |
| γ° | 120 |
| Volume(Å^3^) | 3716.6(6) |
| Z | 6 |
| ρ_calc_ (g/cm^3^) | 1.474 |
| μ (mm^-1^) | 1.601 |
| F (000) | 1680.0 |
| Theta(max) | 27.465 |
| R(reflections) | 0.0408(781) |
| wR2(reflections) | 0.1421(945) |
| no. of parameters | 59 |
| S | 1.151 |

**Table S4. Single crystal X-ray diffraction data of ((C_2_H_5_)_2_NH_2_)_3_InCl_6_ single crystals at 330K.**


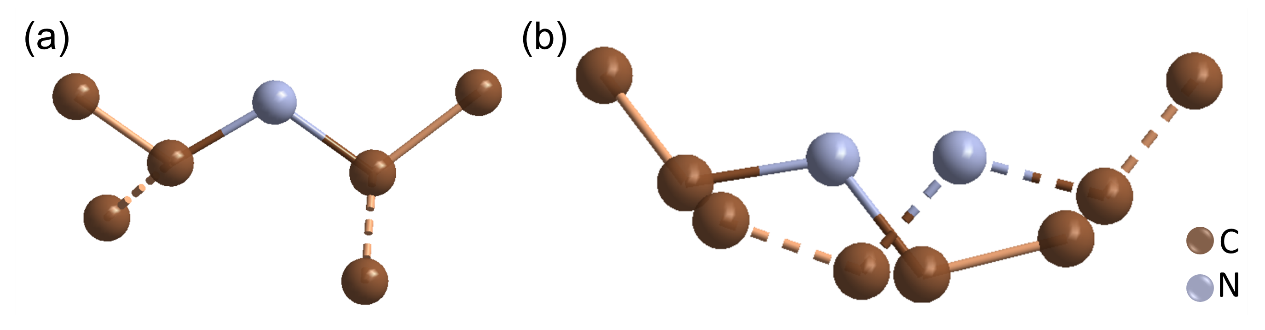


**Figure S1.** The two disordered states of the [(C_2_H_5_)_2_NH_2_]^+^ cation (the hydrogen atom is omitted). Disordered State 1 (a): Order-disorder type phase transition. This transition involves a collapse process where atomic occupation shifts from all possible sites to specific ones.^1^

Disordered State 2: (b) Displacive type phase transition. This transition is characterized by small atomic displacements that result in a change in the symmetry or structure of the material.^1^


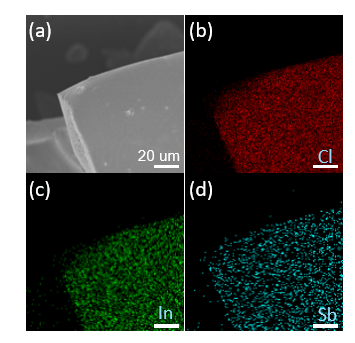


**Figure S2.** SEM image of Sb^3+^-doped ((C_2_H_5_)_2_NH_2_)_3_InCl_6_ (a) and corresponding EDS mappings of (b) Cl, (c) In and (d) Sb elements.


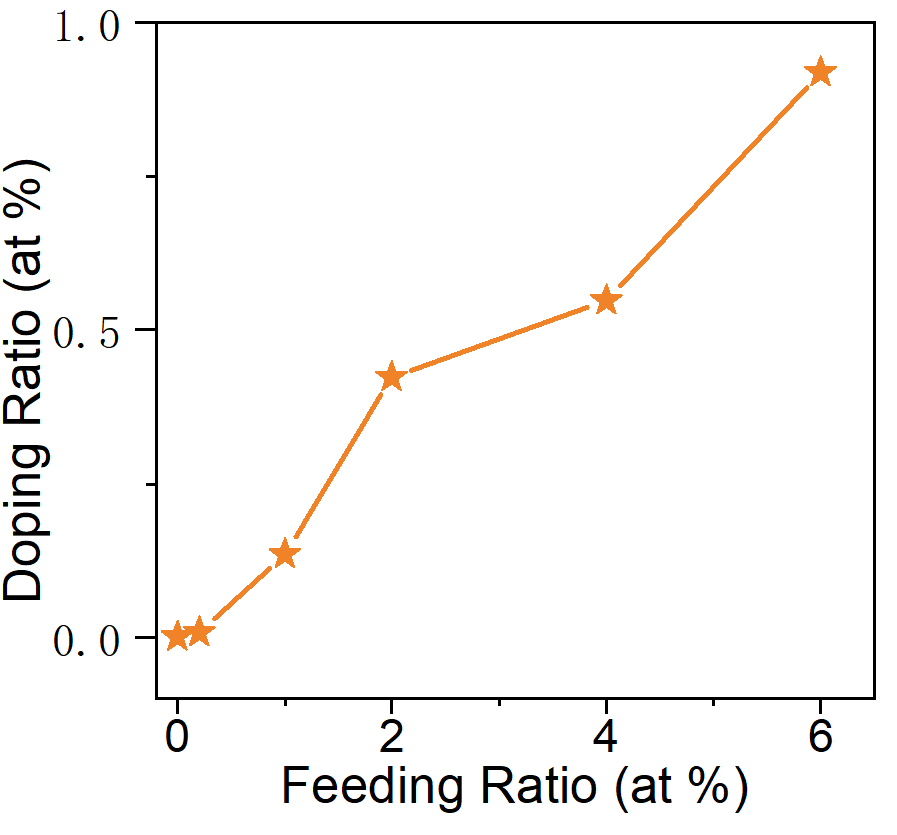


**Figure S3.** ICP results of Sb^3+^ feed amount and doping amount in ((C_2_H_5_)_2_NH_2_)_3_InCl_6_ (atomic ratio).

**
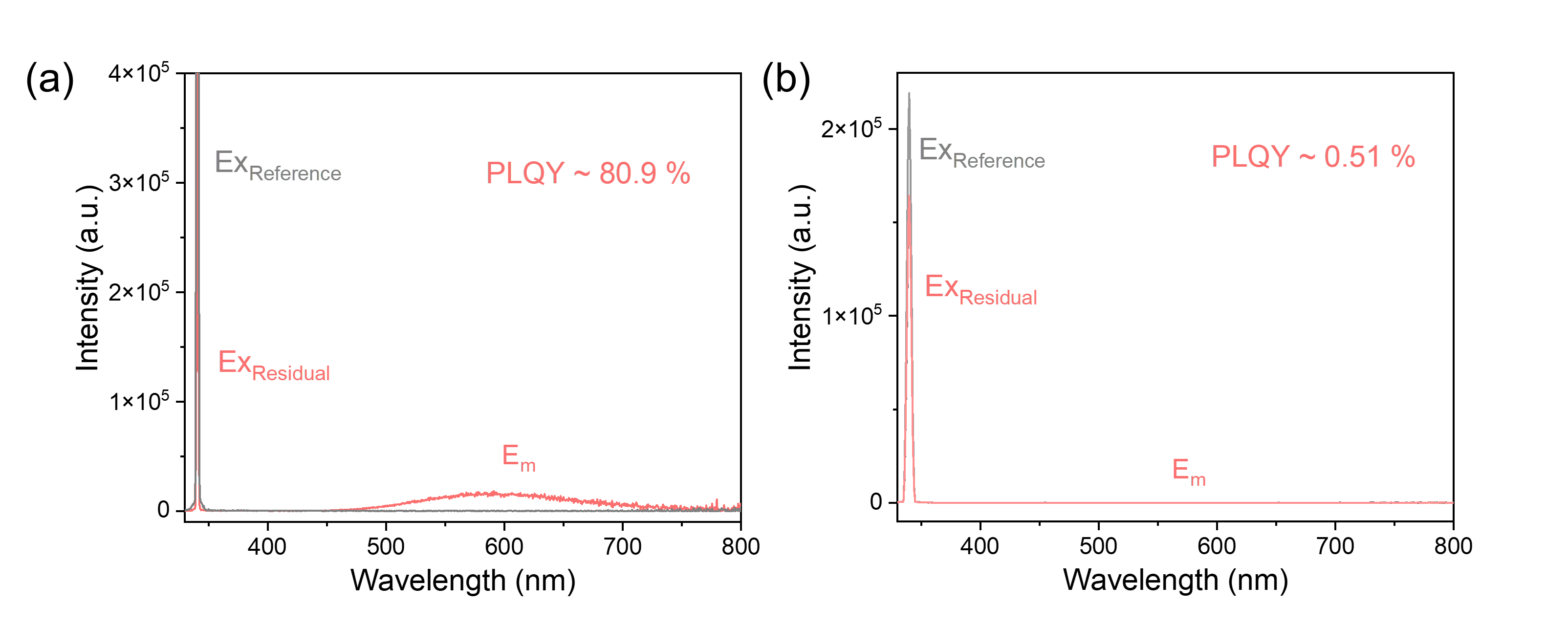
**

**Figure S4.** PL spectra for PLQY of (a) ((C_2_H_5_)_2_NH_2_)_3_InCl_6_:0.42% Sb^3+^ and (b) undoped ((C_2_H_5_)_2_NH_2_)_3_InCl_6_ samples at room temperature.


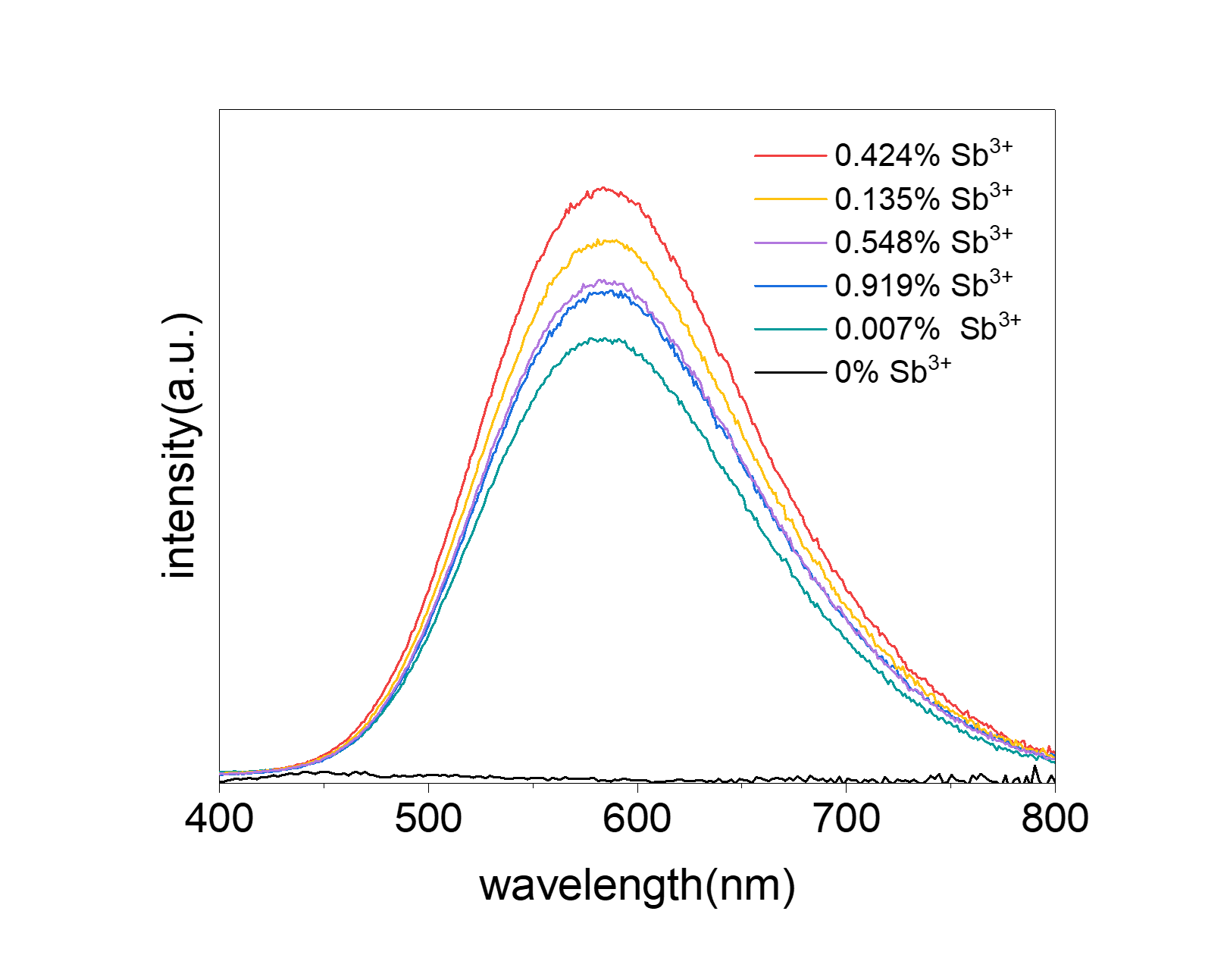


**Figure S5.** PL spectra monitored at 282 nm of x% Sb^3+^:((C_2_H_5_)_2_NH_2_)_3_InCl_6_.


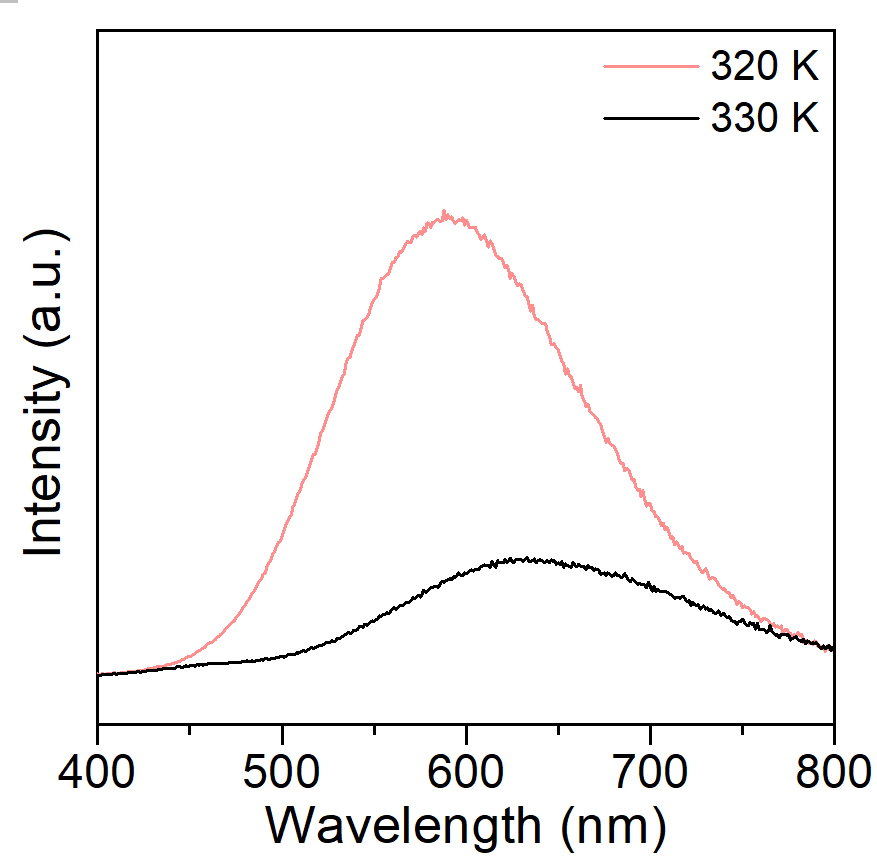


**Figure S6.** PL spectra of ((C_2_H_5_)_2_NH_2_)_3_InCl_6_:0.42%Sb^3+^ at 320 K and 330 K.


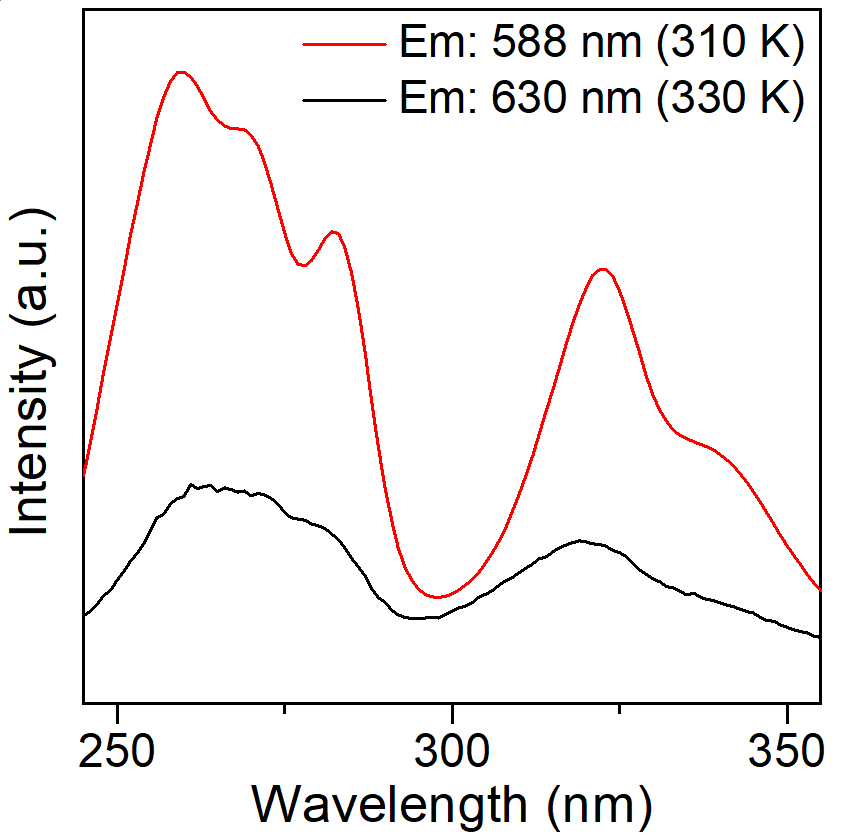


**Figure S7.** PLE spectra of ((C_2_H_5_)_2_NH_2_)_3_InCl_6_:0.42% Sb^3+^ at 310 K and 330 K.


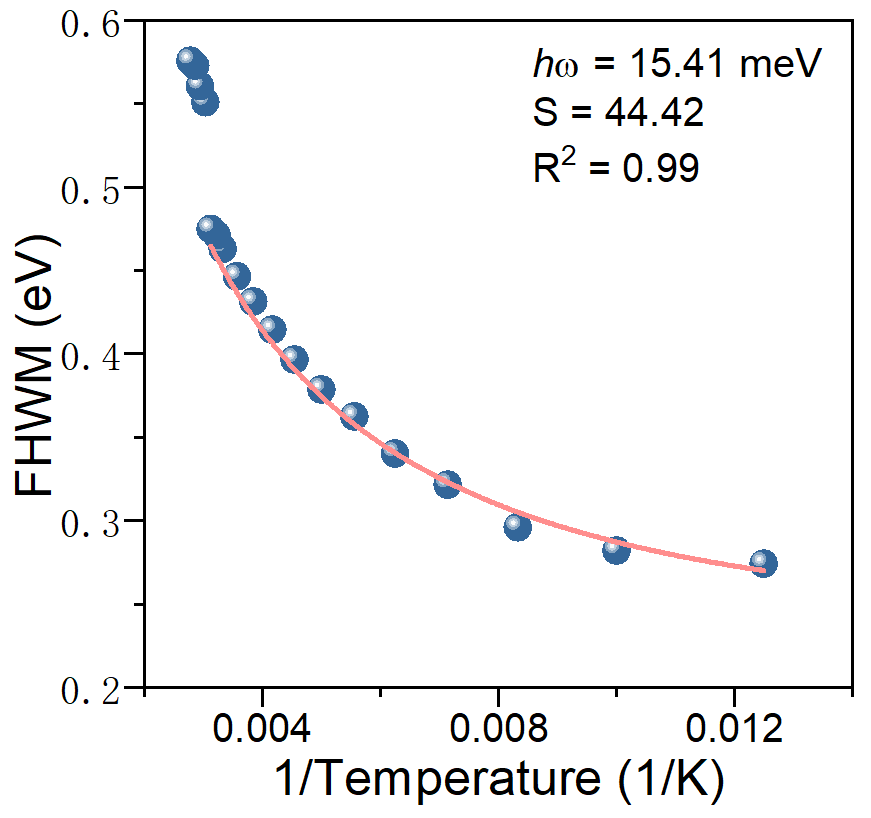


**Figure S8.** FWHM of PL spectra measured from 80 K to 360 K.


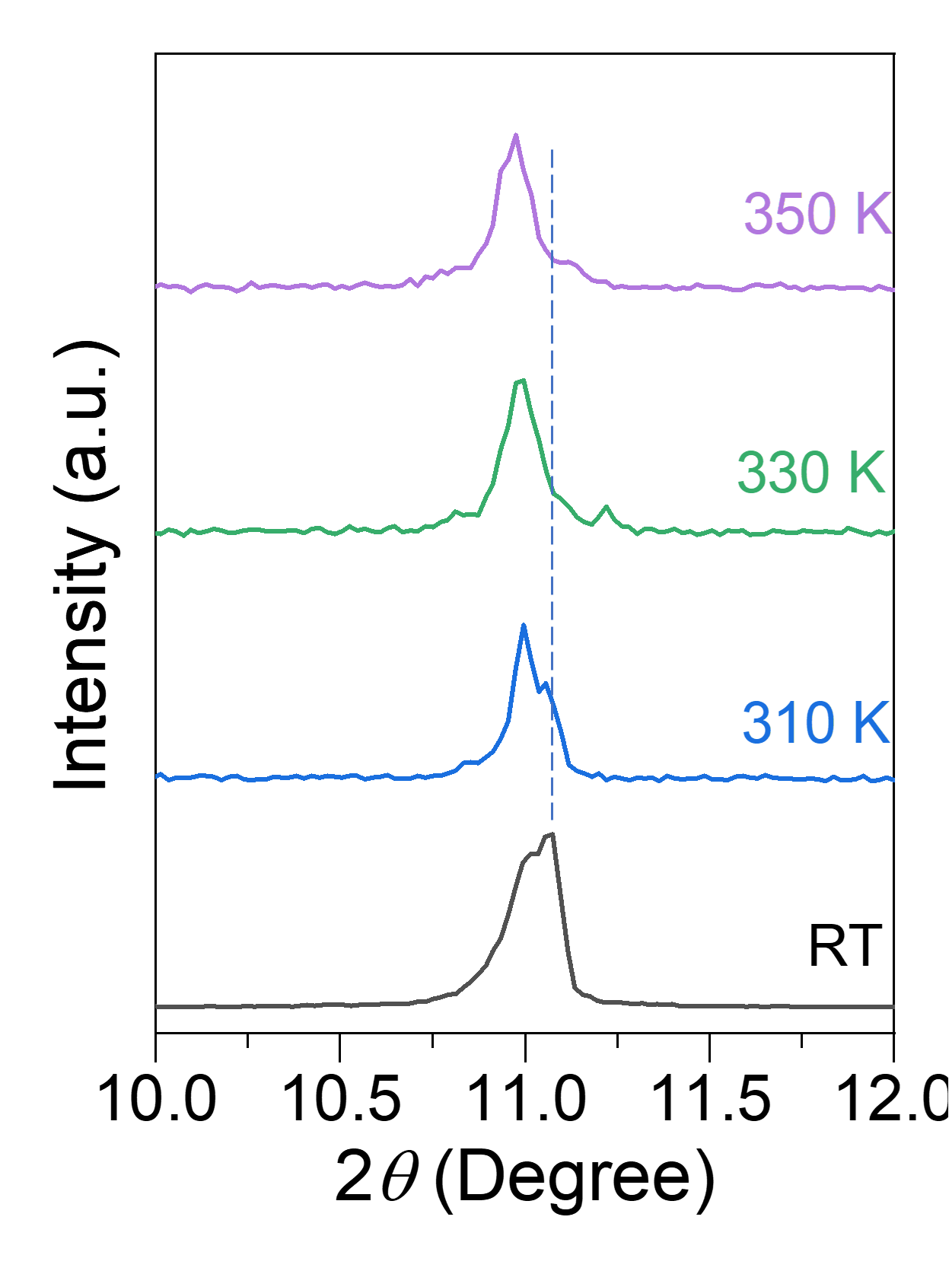


**Figure S9.** Partially magnified temperature-dependent XRD patterns of ((C_2_H_5_)_2_NH_2_)_3_InCl_6_ between 10° and 12°.

**Figure S10.** The DSC curve of heating and cooling processes of Sb^3+^ doped ((C_2_H_5_)_2_NH_2_)_3_InCl_6_.


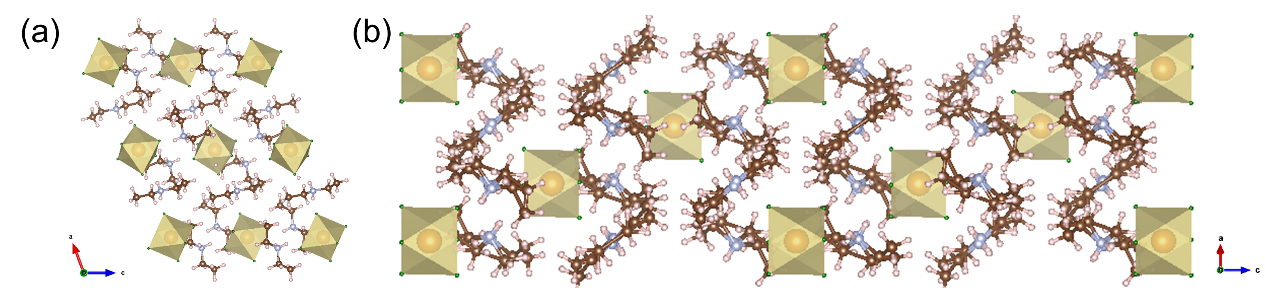


**Figure S11.** Diagram of the crystal structure of ((C_2_H_5_)_2_NH_2_)_3_InCl_6_ at (a) 134 K and (b) 330 K.

**Reference**

(1) X. Wei, N. Domingo, Y. Sun, N. Balke, R. E. Dunin‐Borkowski, J. Mayer, *Adv. Energy Mater.* **2022**, *12* (24), 2201199.
